# Neutropenia Associated With Furosemide Administration in a Preterm Infant

**DOI:** 10.1155/crpe/1141800

**Published:** 2025-11-10

**Authors:** Gillean K. Connolly, Jennifer Keates-Baleeiro, Valerie Curren, Tammy Brady, David Procaccini, John D. Coulson

**Affiliations:** ^1^Department of Pediatrics, Johns Hopkins University School of Medicine, Baltimore, Maryland, USA; ^2^Division of Pediatric Hematology, Johns Hopkins University School of Medicine, Baltimore, Maryland, USA; ^3^Division of Pediatric Cardiology, Johns Hopkins University School of Medicine, Baltimore, Maryland, USA; ^4^Division of Pediatric Nephrology, Johns Hopkins University School of Medicine, Baltimore, Maryland, USA; ^5^Department of Pharmacy, Nemours Children's Hospital, Wilmington, Delaware, USA

## Abstract

Furosemide is a commonly used diuretic, though there is scant literature describing its association with leukopenia. We present a case of neutropenia observed in a preterm infant with a congenital heart anomaly, who received both oral and intravenous furosemide over the course of two hospitalizations. Using the Naranjo adverse drug reaction probability scale, we posit that furosemide was the probable cause of this observed hematologic trend; other etiologies of neutropenia, such as infection, allergy, or physiologic nadir, are unlikely to explain the neutropenia observed in our patient.

## 1. Introduction

There is scant literature describing an association between furosemide and leukopenia. Blood dyscrasias have been reported as possible adverse reactions; however, the frequency and causality of these effects are not described [1]. We observed decreases in neutrophil percentage and absolute neutrophil count (ANC) after both oral and intravenous (IV) furosemide administration in a preterm infant. We propose that these hematologic changes may have been secondary to furosemide. We aim to chronologically describe the observed hematologic trends in our patient in relation to furosemide dosing, discuss why these observed trends were probably caused by furosemide utilizing the Naranjo adverse drug reaction probability scale [2], and describe possible mechanisms of action conferring drug-induced neutropenia.

## 2. Case Presentation

**Figure 1:**
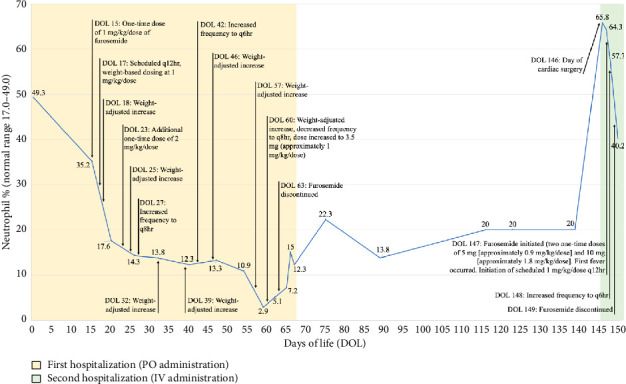
Neutrophil count in association with oral (PO) and intravenous (IV) furosemide administration.

Our patient was born at 32-3/7 weeks of gestation. Echocardiography demonstrated large conoventricular and posterior muscular ventricular septal defects (VSDs), leading to congestive heart failure. An oral dose of furosemide was administered on day of life (DOL) 15; scheduled oral furosemide was initiated on DOL 17 with incrementally increased dosing. Concurrently, neutrophil count declined, ultimately reaching an ANC of 190 on DOL 59. Furosemide was subsequently discontinued on DOL 63 due to concern for possible association with neutropenia. Following furosemide discontinuation, neutrophil counts recovered with an ANC of 780 on DOL 67 (Figure 1).

Approximately 2 months later, our patient was rehospitalized for VSD closure. The postoperative course was complicated by low urine output, which improved with IV furosemide. A decrease in neutrophils was again observed (65.8% on DOL 146 to 40.2% on DOL 150). Our patient's ANC decreased from 7580 on DOL 146 to 6500 on DOL 147, rose to 9600 on DOL 148, and then decreased to 3310 on DOL 150.

The Naranjo adverse drug reaction probability scale was utilized to assess the causality of the effect [2]. Our patient's score (5) indicates that furosemide was probably the cause of the neutropenia observed in our patient (Table 1) [2].

## 3. Discussion

This case suggests an association between decreased neutrophil counts and furosemide administration during one or perhaps two hospitalizations. It is unlikely that alternative explanations, including drug–drug interactions, physiologic nadir, or infection, explain the degree of neutropenia observed. Although our patient received drugs that have established interactions with furosemide (captopril, hydrochlorothiazide, and chlorothiazide), the associated adverse effects include electrolyte abnormalities, hypotension, nephrotoxicity, and enhanced pharmacodynamic effects, but not blood dyscrasias. Neonates normally have a downtrending ANC, decreasing to “a lower ANC limit of 1000/μL until 1 year of age” [3]; however, our patient's ANC decreased far below this lower limit with an ANC nadir of 190, suggesting a nonphysiologic process. Abnormal neutrophil morphology was not observed on peripheral blood smears. Infection is an established cause of neutropenia [4]; however, there was no concern for infection during the first hospitalization with no antibiotics administered. During a second hospitalization for surgery, our patient developed fever with elevations in white blood cell count (16.68 K/cu mm) and C-reactive protein (11.4 mg/dL) and received antibiotics (perioperative cefazolin for two doses postsurgery, followed by cefepime for 2 days after fever). However, blood culture and respiratory viral panel were negative.

Literature regarding the effects of furosemide on different cell types is sparse and varied. Agranulocytosis [5–7], thrombocytopenia [8, 9], aplastic anemia [10], and leukopenia [11] have been associated with furosemide administration. Another study did not find furosemide to increase the risk of agranulocytosis [8]. One case-control study found furosemide to increase the risk of agranulocytosis (relative risk [RR] 2.0) when adjusted for geographic area, age, and sex [10]. This increased risk was diminished after adjustment for the simultaneous use of other drugs (RR 0.9, 95% CI 0.4–2.0) [10]. Others found neutropenia to be an infrequent side effect of furosemide among adults [12]. Literature review revealed only one case report describing episodes of leukopenia and neutropenia in a pediatric patient after receiving furosemide [13]. The author noted brief decreases in leukocyte counts with furosemide in a preterm neonate [13]. The infant's leukocytes increased appropriately within 24–48 h after furosemide doses, suggesting that the effect was on peripheral white blood cells and not the bone marrow, whose function remained intact [13].

Other possible adverse effects of furosemide in neonates include electrolyte derangements and dehydration [14]. Furosemide can also facilitate opening of the patent ductus arteriosus due to increased production of prostaglandin E2 [14]. Furosemide may lead to ototoxicity (including hearing loss) and nephrolithiasis, and preterm or low birth weight infants are at risk of nephrocalcinosis [14]. However, more recent studies have found limited evidence demonstrating risk of sensorineural hearing loss, nephrocalcinosis, or nephrolithiasis associated with furosemide use among preterm neonates [15]; one randomized clinical trial found no increased incidence of hearing loss, nephrocalcinosis, or nephrolithiasis among premature infants receiving furosemide [16].

Furosemide is a chlorobenzoic acid sulfonamide, which possesses a sulfa-group within its chemical structure. Sulfonamide medications are known to be associated with cytopenias [9], including agranulocytosis [17] and thrombocytopenia [18]. The chemical structure shared with other sulfonamides might explain leukopenia observed with furosemide [13]. Two mechanisms have been proposed for sulfonamide-induced agranulocytosis [19]. One posits an immune-mediated response in which neutrophils are destroyed by antibodies that form with drug exposure [19, 20]. The second mechanism proposes direct bone marrow toxicity secondary to oxidative by-products of drug metabolism leading to destruction of myeloid cells [19, 20]. Interestingly, furosemide does not contain an aromatic amine group, which is thought to cause allergic reactions associated with sulfonamide antibiotics [21]. Patients with allergies to sulfonamides are often able to take furosemide due to differences in drug metabolism [21]. This drug tolerance makes it unlikely for an allergic-mediated or generalized cross-reactive process to confer adverse effects observed with furosemide [22].

Decreases in neutrophil counts were observed in our patient with both oral and IV administration. Bioavailability of orally administered furosemide varies greatly, typically around 50%, but ranging between 10% and 90% in one report [23] and between 37% and 51% in another [24]. Individual differences in bioavailability may be due to variations in gastric emptying [24]. Additionally, oral furosemide has increased bioavailability if administered before eating, as food interferes with absorption [23]. In contrast, IV furosemide has low variability in mean residence time among individuals [24] with a faster onset of action compared to oral furosemide [25]. In an adult case report, leukopenia in a 72-year-old man observed during multiple hospitalizations seemed to be associated with IV furosemide. Interestingly, this patient was receiving a lower dose of oral furosemide at home without sustained leukopenia, suggesting a dose-related and/or concentration-dependent effect with IV administration [26].

Given the wide variability in oral bioavailability of furosemide, it is interesting that our patient developed neutropenia with oral dosing, particularly in light of their feeding schedule, which included continuous feeds (via nasogastric or nasoduodenal tubes) before transitioning to frequent bolus feeds. Thus, there were likely many days during which feeds interfered with furosemide bioavailability, suggesting (1) neutropenia may not be a dose-dependent effect of furosemide and/or (2) that neonatal patients are more susceptible to this effect with the use of furosemide despite the route of administration. Alternative mechanisms, such as genetic susceptibility impacting pharmacokinetics or an exposure-dependent response, could be responsible for neutropenia.

The development of severe neutropenia (ANC < 500/μL) has clinically significant implications, including increased risk of nosocomial infection in neonates [27]. In addition to concerns for sepsis, early-onset neutropenia among preterm neonates can also be associated with periventricular hemorrhage, asphyxia, or growth restriction [28]. Neonatal neutropenia may also suggest the presence of additional complications, such as bone marrow suppression, hemolytic disease, or necrotizing enterocolitis [27].

Premature infants are at higher risk of neutropenia because most neutrophils are formed in the final trimester of pregnancy [3, 29]. Gestational age correlates positively with absolute cell counts, suggesting greater leukopenia among more premature infants [30]. Additionally, although neutropenia is not uncommon among preterm neonates (with rates of occurrence between 6% and 58%) [27], higher rates of neutropenia have been observed in patients with a VSD compared to patients with acyanotic congenital cardiac disease without a VSD [31]. This observed association between VSDs and neutropenia may be secondary to various embryologic factors [31]. Our patient was born at 32-3/7 weeks of gestation, so prematurity, in addition to the presence of a VSD, may have contributed to neutropenia. Because the second hospitalization was shorter with only a few days of furosemide administration, it is difficult to trend neutrophil counts and ANC. Additionally, the observed neutrophil decline during this postoperative period may be solely attributable to possible infection manifested by fever and/or inflammation after surgery. Our patient was receiving other medications concurrently with furosemide, which might have contributed to neutropenia. During the first hospitalization, our patient received captopril, which can be associated with drug-induced agranulocytosis [5, 7], and hydrochlorothiazide, part of the thiazide class of medications with known associations with agranulocytosis [7]. Cefazolin and cefepime were administered during the second hospitalization; cephalosporins have known associations with agranulocytosis [7].

## 4. Conclusion

Use of furosemide in the reported neonatal patient was associated with neutropenia, which has important clinical implications. The high utilization of this medication, including within pediatric populations, underscores the need for heightened clinical awareness. Further investigation to determine the clinical significance is warranted before altering recommendations regarding furosemide administration and/or dosing in preterm neonates.

## Figures and Tables

**Table 1:** Naranjo adverse drug reaction probability scale [2].

Question	Yes	No	Do not know	Score
Are there previous conclusive reports on this reaction?	+1	0	0	+1
Did the adverse event appear after the suspected drug was administered?	+2	−1	0	+2
Did the adverse reaction improve when the drug was discontinued or a specific antagonist was administered?	+1	0	0	+1
Did the adverse reaction reappear when the drug was readministered?	+2	−1	0	0
Are there alternative causes (other than the drug) that could on their own have caused the reaction?	−1	+2	0	−1
Did the reaction reappear when a placebo was given?	−1	+1	0	0
Was the drug detected in the blood (or other fluids) in concentrations known to be toxic?	+1	0	0	0
Was the reaction more severe when the dose was increased, or less severe when the dose was decreased?	+1	0	0	+1
Did the patient have a similar reaction to the same or similar drugs in any previous exposures?	+1	0	0	0
Was the adverse event confirmed by any objective evidence?	+1	0	0	+1

*Note:* Total score: 5. Score ≥ 9: definite adverse drug reaction (ADR). Scores 5 to 8: probable ADR. Scores 1 to 4: possible ADR. Scores ≤ 0: doubtful ADR.

## Data Availability

All data used in the study are available in the published literature.
